# Supplementary material for: Interaction of a dinoflagellate neurotoxin with voltage-activated ion channels in a marine diatom
Source: PeerJ. 2018 Apr 3;6:e4533. doi: 10.7717/peerj.4533 (PMC5888156; doi:10.7717/peerj.4533)
Supplement: Figure S2 — Multiple sequence alignment of predicted VGCs proteins was produced using MUSCLE in Geneious v8.0.3. The protein alignment was truncated at the N- and C-terminal ends to the conserved sequence region. Characterized transmembrane segments (S1–6, red bars) and the ascending and descending helices (P1–2, purple bars) comprising the pore domain are indicated under the NaChBac sequence. The selectivity filter referenced in Fig. 4B is shown within the pore domain, inside the green box. Sequence similarity was determined using a Blosum62 score matrix (Henikoff & Henikoff, 1992) where > 60% is white, 60–80% is light grey, 80 to 99% is dark grey, and 100% is black. [file peerj-06-4533-s002.pdf]

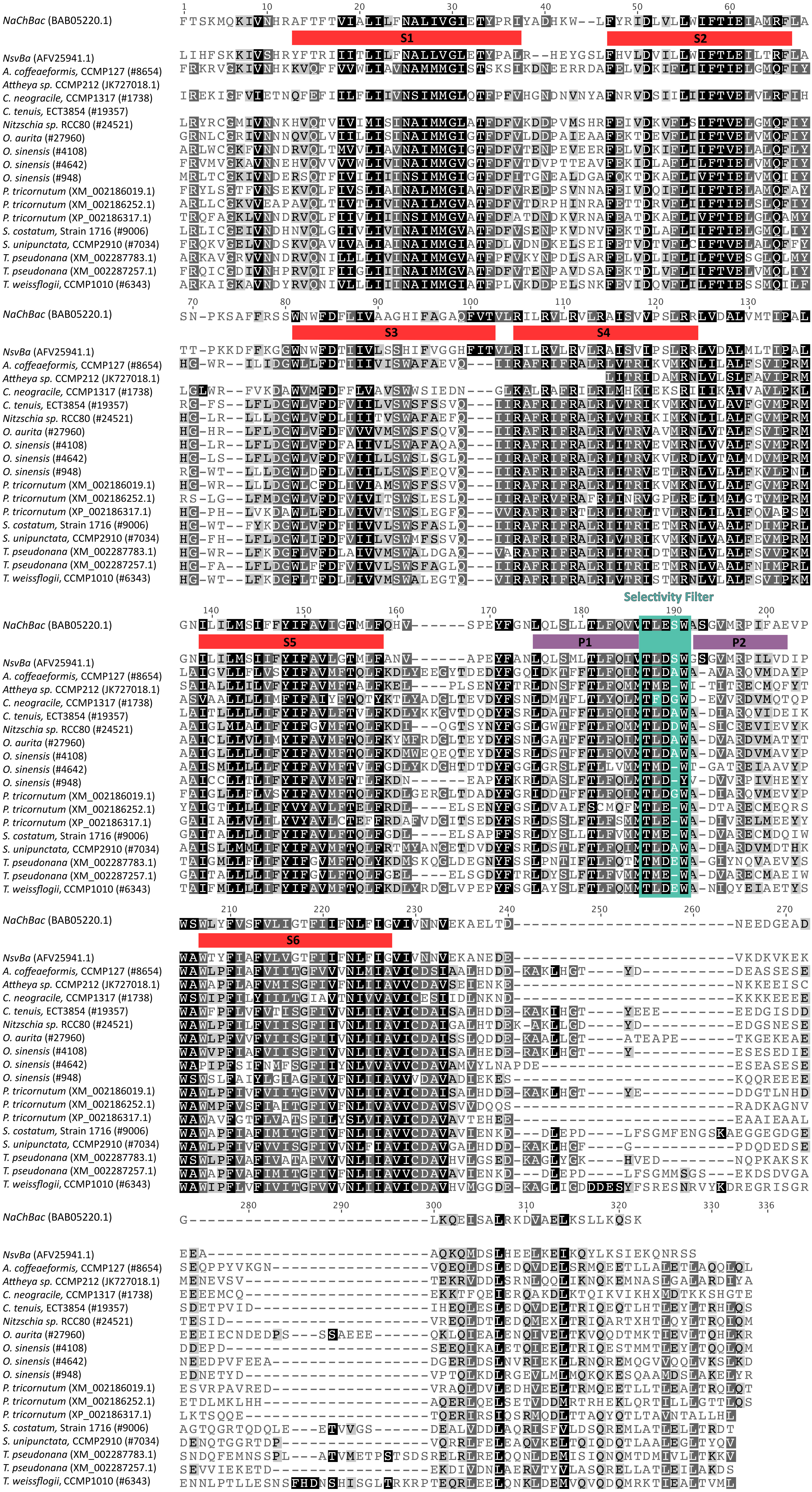
NaChBac (BAB05220.1)
NsvBa (AFV25941.1)
A. coffeaeformis, CCMP127 (#8654)
Attheya sp. CCMP212 (JK727018.1)
C. neogracile, CCMP1317 (#1738)
C. tenuis, ECT3854 (#19357)
Nitzschia sp. RCC80 (#24521)
O. aurita (#27960)
O. sinensis (#4108)
O. sinensis (#4642)
O. sinensis (#948)
P. tricornutum (XM_002186019.1)
P. tricornutum (XM_002186252.1)
P. tricornutum (XP_002186317.1)
S. costatum, Strain 1716 (#9006)
S. unipunctata, CCMP2910 (#7034)
T. pseudonana (XM_002287783.1)
T. pseudonana (XM_002287257.1)
T. weissflogii, CCMP1010 (#6343)
S1
S2
S3
S4
S5
S6
P1
P2
Selectivity Filter
1 10 20 30 40 50 60 70 80 90 100 110 120 130 140 150 160 170 180 190 200 210 220 230 240 250 260 270 280 290 300 310 320 330 336
